# Supplementary material for: Conditional chemoconnectomics (cCCTomics) as a strategy for efficient and conditional targeting of chemical transmission
Source: eLife. 2024 Apr 30;12:RP91927. doi: 10.7554/eLife.91927 (PMC11060718; doi:10.7554/eLife.91927)
Supplement: Figure 3—source data 3. [file elife-91927-fig3-data3.zip › Figure 3 source data3 part 2.zip!/Figure 3-Source data 3-part2/1062-13-CRISPResso_Report_jt28fhr9.zip!/CRISPResso_on_1062_16/2a.gcaggaaatgagctacctac.Nucleotide_percentage_quilt.pdf]

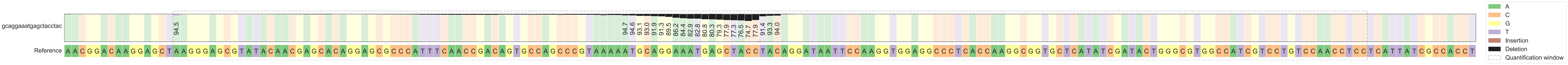

gcaggaaatgagctacctac
94.5
94.7
94.6
93.1
93.0
91.9
91.3
89.5
86.2
84.4
82.9
82.8
80.8
80.3
79.3
77.9
77.3
76.5
74.7
77.9
91.4
93.3
94.0
Reference
AACGGACAAGGAGCTAAGGGAGCGTATACAACGAGCACAGGAGCGCCCATTTCAACCGACAGTGCCAGCCCGTAAAAATGCAGGAAATGAGCTACCTACAGGATAATTCCAAGGTGGAGGCCCTCACCAAGGCGGTGCTCATATCGATACTGGGCGTGGCCATCGTCCTGTCCAACCTCCTCATTATCGCCACCT
A
C
G
T
Insertion
Deletion
Quantification window
